# Apoptosis of Kinetin Riboside in Colorectal Cancer Cells Occurs by Promoting β-Catenin Degradation

**DOI:** 10.4014/jmb.2301.01035

**Published:** 2023-06-23

**Authors:** TaeKyung Nam, Wonku Kang, Sangtaek Oh

**Affiliations:** 1 Department of Bio and Fermentation Convergence Technology, Kookmin University, Seoul 02707, Republic of Korea; 2 College of Pharmacy, Chung-Ang University, Seoul 06974, Republic of Korea

**Keywords:** Kinetin riboside, Wnt/β-catenin pathway, protein degradation, colorectal cancer, apoptosis

## Abstract

Kinetin riboside is a naturally produced cytokinin that displays strong antiproliferative activity in various human cancer cells. However, the mechanism of chemoprevention in colorectal cancer cells has not been elucidated. We used a cell-based reporter system to identify kinetin riboside as an antagonist of the Wnt/β-catenin pathway, which is aberrantly upregulated in colorectal cancer. Kinetin riboside suppressed β-catenin response transcription (CRT) by accelerating the degradation of intracellular β-catenin via a proteasomal degradation pathway. Pharmacological inhibition of glycogen synthase kinase-3β did not affect CRT downregulation. Kinetin riboside decreased the intracellular β-catenin levels in colorectal cancer cells with mutations in adenomatous polyposis coli (APC) and β-catenin. Consistently, kinetin riboside repressed expression of c-Myc and cyclin D1, β-catenin/T-cell factor (TCF)-dependent genes, and inhibited the proliferation of colorectal cancer cells. In addition, kinetin riboside stimulated apoptosis, as measured by an increase in annexin V-FITC-stained cells. These findings suggest that kinetin riboside exerts its anti-cancer activity by promoting β-catenin degradation and has significant potential as a chemopreventive agent for colorectal cancer cells.

## Introduction

The Wnt/β-catenin pathway plays essential roles in regulating various cellular behaviors, including proliferation, survival, and differentiation [1-3]. The intracellular β-catenin level, which is regulated by a proteasomal degradation pathway, is critical to Wnt/β-catenin pathway control [4]. Normally, casein kinase 1 (CK1) and glycogen synthase kinase-3β (GSK-3β), which form a complex with the scaffolding protein Axin and the tumor suppressor protein adenomatous polyposis coli (APC), phosphorylate β-catenin at Ser45, Thr41, Ser37, and Ser33 [5, 6]. Phosphorylated β-catenin is ubiquitinated by the β-transducin repeat-containing protein (β-TrCP), an F-box E3 ubiquitin ligase complex, and ubiquitinated β-catenin is degraded via a proteasome pathway [7, 8].

Colorectal cancer is a significant cause of cancer-related deaths worldwide. Abnormal up-regulation of the Wnt/β-catenin pathway is a major pathological event in intestinal epithelial cells during human colorectal cancer oncogenesis [9]. Genetic mutations in the *APC* gene are observed in familial adenomatous polyposis coli (FAP) and sporadic colorectal cancers [10]. In addition, mutations in the N-terminal phosphorylation motif of the β-catenin gene were found in patients with colorectal cancer [11]. These mutations cause β-catenin to accumulate in the nucleus, where it forms complexes with transcription factors of the T-cell factor/lymphocyte enhancer factor (TCF/LEF) family to stimulate the expression of β-catenin responsive genes, such as *c-Myc* and *cyclin D1*, which leads to colorectal tumorigenesis [12-14]. Therefore, downregulating β-catenin response transcription (CRT) is a potential strategy for preventing and treating colorectal cancer.

Plant cytokinins are *N*^6^-substituted purine derivatives; they promote cell division in plants and regulate developmental pathways. Natural cytokinins are classified as isoprenoid (isopentenyladenine, zeatin, and dihydrozeatin), aromatic (benzyladenine, topolin, and methoxytopolin), or furfural (kinetin and kinetin riboside), depending on their structure [15, 16]. Kinetin riboside was identified in coconut water and is a naturally produced cytokinin that induces apoptosis and exhibits antiproliferative activity in several human cancer cell lines [17]. However, little attention has been paid to kinetin riboside’s mode of action. In this study, we show that kinetin riboside exerts its cytotoxic activity against colon cancer cells by suppressing the Wnt/β-catenin pathway and promoting intracellular β-catenin degradation.

## Materials and Methods

### Cell culture, Reporter Assays, and Chemicals

HEK293, SW480, HCT116, and Wnt3a-secreting L cells were obtained from the American Type Culture Collection (USA) and cultured in Dulbecco’s Modified Eagle’s medium (DMEM) containing 1% penicillin/streptomycin and 10% fetal bovine serum (FBS). HEK293-firefly luciferase reporter cells were established as previously described [18]. Wnt3a-conditioned medium (Wnt3a-CM) was made from Wnt3a-secreting L cells. Firefly luciferase assays were performed using Dual Luciferase Assay Kits (Promega, USA) according to the manufacturer’s instructions. 6-Bromoindirubin-3’-oxime (BIO), kinetin riboside, and MG-132 were purchased from Sigma-Aldrich (USA).

### Western Blot Analysis

Cytosolic fractions were prepared as previously described [19]. Proteins were separated by sodium dodecyl sulfate-polyacrylamide gel electrophoresis and transferred to nitrocellulose membranes (Bio-Rad, USA). The membranes were blocked using SuperBlock Blocking Buffer (Thermo Fisher Scientific, USA) for 30 min at room temperature and probed overnight with anti-β-catenin (BD transduction Laboratories, USA), anti-active-β-catenin (Cell signaling Technology, USA), anti-cyclin D1 (ABclonal Technology, USA), anti-c-Myc (ABclonal Technology), anti-PARP (Cell Signaling Technology), anti-caspase-3 (Cell Signaling Technology), anti-cleaved caspase-3 (Cell Signaling Technology), and anti-actin (Cell Signaling Technology) antibodies. Membranes were then incubated with horseradish-peroxidase-conjugated anti-mouse IgG (Santa Cruz Biotechnology, USA) or anti-rabbit IgG (Santa Cruz Biotechnology), and reacted proteins were visualized using an enhanced chemiluminescence system (Santa Cruz Biotechnology).

### Semi-Quantitative RT-PCR

Total RNA was isolated with Trizol reagent (Invitrogen, USA) following the manufacturer’s protocols. cDNA synthesis, reverse transcription, and PCR were performed as described previously [20]. Amplified DNA was stained with ethidium bromide and separated using 2% agarose gel electrophoresis. The forward and reverse primers used to detect the indicated genes were as follows: β-catenin, 5’-GGATGTTCACAACCGAATTGTTATG-3’ and 5’-ACCAGAGTGAAAAGAACGATAGCTAGGA-3’; human glyceraldehyde-3-phosphate dehydrogenase (GAPDH), 5’-ACCACAGTCCATGCCATCAC-3’ and 5’-TCCACCACCCTGTTGCTGTA-3’.

### Cell Viability

SW480 and HCT116 cells (0.5 × 10^4^ cells/well) were seeded into 96-well plates and treated with kinetin riboside for 48 h. Cell viabilities for each treated sample were measured in triplicate using a Celltiter-Glo assay kit (Promega) according to the manufacturer’s instructions.

### Apoptosis Assays

SW480 and HCT116 cells treated with kinetin riboside for 48 h were washed with cold phosphate-buffered saline and stained with annexin V-fluorescein isothiocyanate (FITC) and propidium iodide (PI), using an ApoScanTM annexin V-FITC apoptosis detection kit (BD Transduction Laboratories), according to the manufacturer’s instructions. Apoptosis rates were calculated using a Cellometer Vision Image Cytometer (Nexcelom Bioscience, USA).

### Statistical Analysis

The student’s *t*-test was used to compare the means between the control and experimental groups. All experiments were performed in triplicate. Statistical significance was set at *p* < 0.05 or *p* < 0.01. Results are expressed as mean ± standard deviation (SD).

## Results

### Kinetin Riboside Suppresses the Wnt/β-Catenin Pathway

**Fig. 1:**
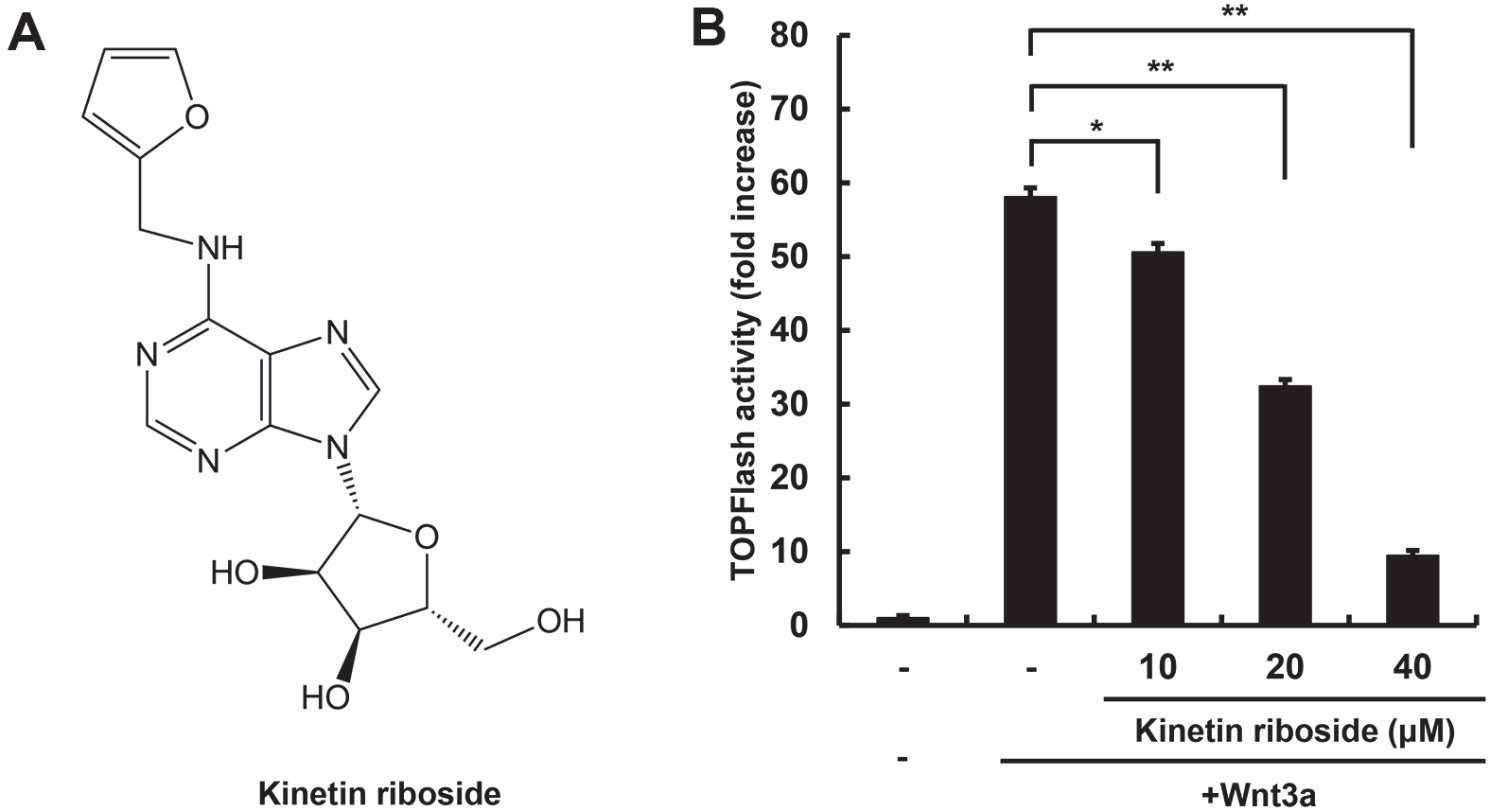
Kinetin riboside antagonizes the Wnt/β-catenin pathway. (**A**) Chemical structure of kinetin riboside. (**B**) HEK293-FL cells were incubated with DMSO or the indicated concentrations of kinetin riboside in the presence of Wnt3a-CM. After 15 h, firefly luciferase activities were detected. Results are expressed as the mean ± SD of three independent experiments. *p < 0.05 and **p < 0.01, comparison between the Wnt3a-CM-treated control and kinetin riboside-treated groups.

We used HEK293-firefly luciferase (FL) reporter cells that were stably transfected with synthetic β-catenin/Tcf-dependent FL reporter gene (TOPFlash) and human Frizzled-1 (hFz-1) expression plasmid (hereafter referred to as HEK293-FL reporter cells) to determine whether kinetin riboside, a purine derivative (Fig. 1A), suppresses the Wnt/β-catenin pathway. After incubating the HEK293-FL reporter cells in Wnt3a-conditioned medium (Wnt3a-CM) and kinetin riboside, FL activity and cell viability were measured using a microplate reader. The effect of kinetin riboside on FL activity was normalized to cell viability. Kinetin riboside treatment decreased the amount of CRT activated by Wnt3a-CM in a dose-dependent manner without detectable cytotoxicity (Figs. 1B and S1), suggesting that kinetin riboside is a specific inhibitor of the Wnt/β-catenin pathway.

### Kinetin Riboside Causes Proteasomal Degradation of β-Catenin

**Fig. 2:**
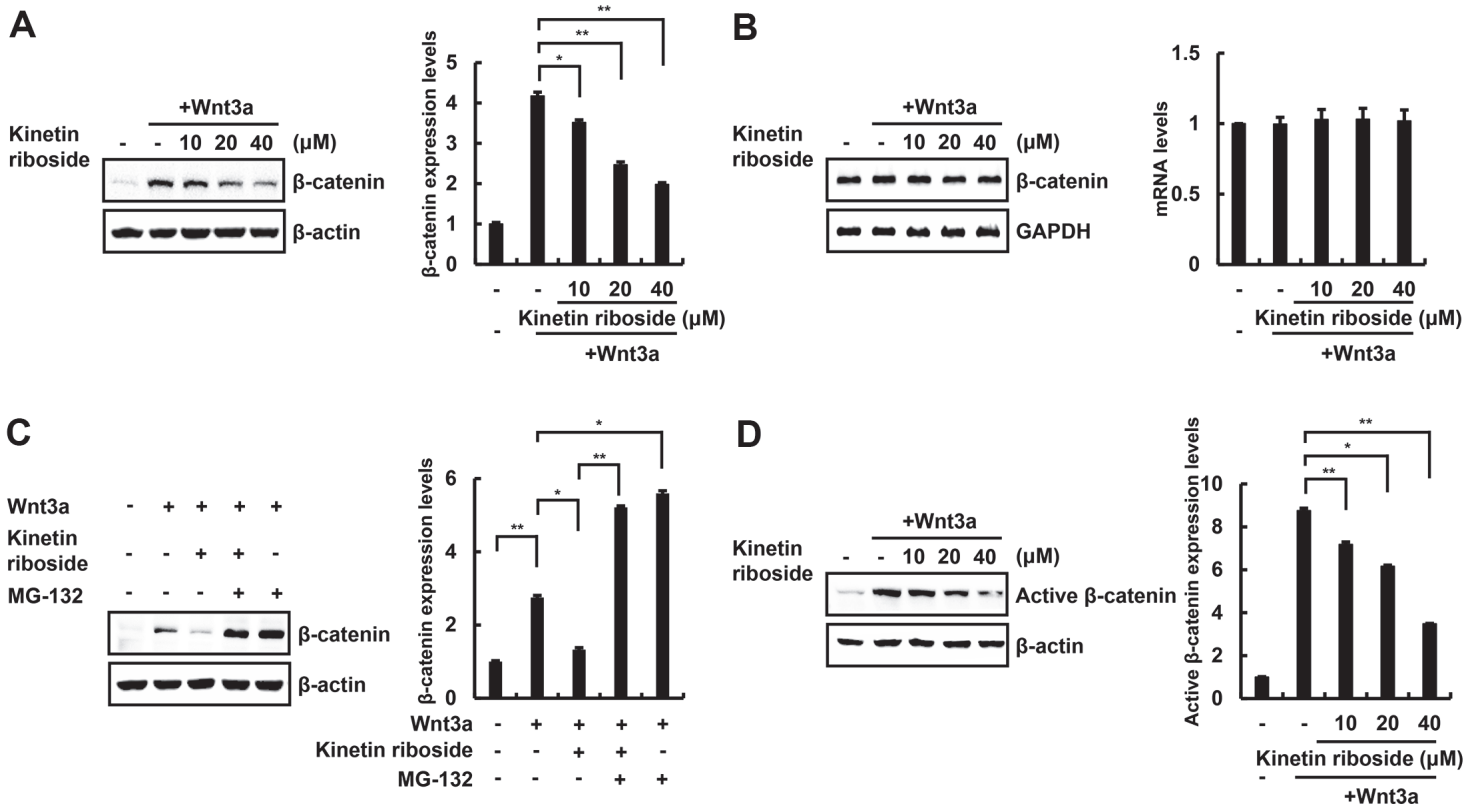
Kinetin riboside promotes proteasomal degradation of β-catenin. (**A**) In the presence or absence of Wnt3a- CM, HEK293-FL cells were treated with vehicle (DMSO) or kinetin riboside (10, 20, or 40 μM) for 15 h, and cytosolic proteins were analyzed by Western blotting with anti-β-catenin antibody. (**B**) In the presence or absence of Wnt3a-CM, HEK293-FL cells were treated with vehicle (DMSO) or kinetin riboside (10, 20, or 40 μM) for 15 h, and β-catenin and GAPDH mRNA levels were detected by semi-quantitative RT-PCR. β-Catenin mRNA levels were normalized to those of GAPDH. (**C**) In the presence or absence of Wnt3a-CM, HEK293-FL cells were incubated with vehicle (DMSO) or kinetin riboside (20 μM) and then exposed to MG-132 (10 μM) for 8 h. Cytosolic proteins were analyzed by Western blotting with anti-β-catenin antibodies. (**D**) After treatment of HEK293-FL cells with DMSO or kinetin riboside (10, 20, or 40 μM) for 15 h, cytosolic proteins were analyzed by Western blotting with anti-active-β-catenin antibody. In (**A**), (**C**), and (**D**), β-actin was used as a loading control, and β-catenin levels were normalized to those of β-actin. The bar graph indicates the average volume density corrected for the loading control, and results are expressed as the mean ± SD of three independent experiments. *p < 0.05 and **p < 0.01, comparison between the Wnt3a-CM-treated control and kinetin riboside-treated groups.

The effect of kinetin riboside on intracellular levels of β-catenin protein was investigated by Western blot analysis with anti-β-catenin antibody. Consistent with the effects of kinetin riboside on CRT, incubating HEK293-FL reporter cells with different concentrations of kinetin riboside attenuated the Wnt3a-CM-mediated increase in intracellular β-catenin protein levels in a dose-dependent manner (Fig. 2A). In contrast, the amount of β-catenin mRNA was not affected by kinetin riboside treatment at any of the concentrations used (Fig. 2B).

MG-132, a proteasome inhibitor, was used to examine the involvement of proteasomes in the kinetin riboside-induced downregulation of β-catenin protein. Kinetin riboside treatment consistently decreased intracellular β-catenin levels in HEK293-FL reporter cells; however, the addition of MG-132 nullified kinetin riboside-mediated β-catenin downregulation (Fig. 2C). Because phosphorylation at Ser33/37/Thr41 of β-catenin is crucial for its proteasomal degradation, we tested the effect of kinetin riboside on β-catenin phosphorylation using Western blot analysis. As shown in Fig. 2D, levels of non-phosphorylated Ser33/37/Thr41 (indicating active β-catenin) were lowered by kinetin riboside. These findings suggest that kinetin riboside attenuates the Wnt/β-catenin pathway by promoting proteasome-mediated degradation of β-catenin.

### Kinetin Riboside Induces β-Catenin Degradation via a GSK-3β-Independent Mechanism

**Fig. 3:**
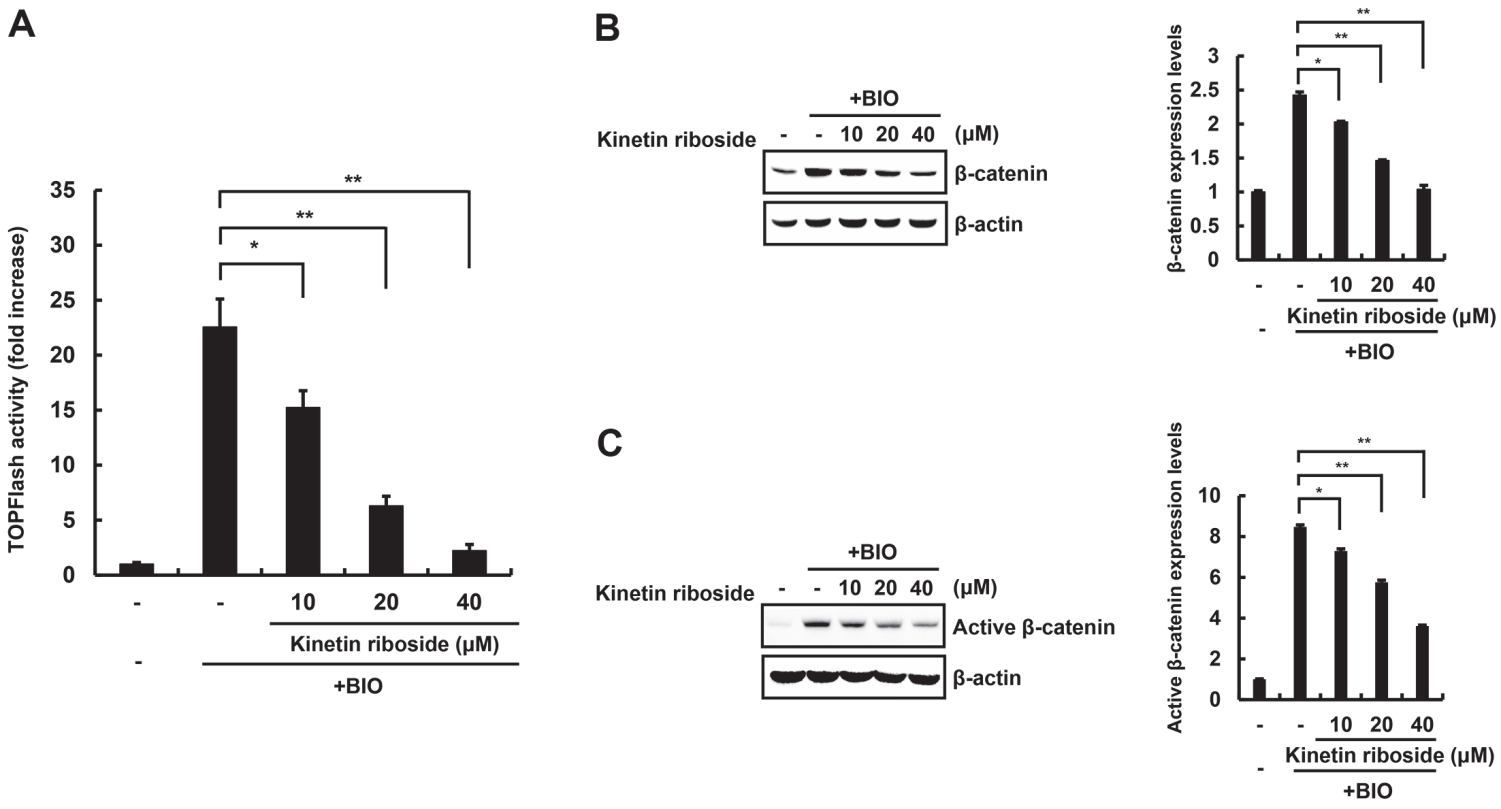
Kinetin riboside stimulates β-catenin phosphorylation and degradation via a mechanism independent of GSK-3β. (**A**) HEK293-FL cells were treated with vehicle (DMSO) or kinetin riboside (10, 20, or 40 μM) in the presence of 0.75 μM BIO for 15 h, and FL activity was measured. Results are expressed as the mean ± SD of three independent experiments. *p < 0.05 and **p < 0.01, comparison between the BIO-treated control and kinetin riboside-treated groups. (**B**) HEK293-FL cells were treated with vehicle (DMSO) or kinetin riboside (10, 20, or 40 μM) in the presence of 0.75 μM BIO for 15 h. Cytosolic proteins were analyzed by Western blotting with anti-β-catenin antibody. (**C**) HEK293-FL cells were treated with vehicle (DMSO) or kinetin riboside (10, 20, or 40 μM) in the presence of 0.75 μM BIO for 15 h. Cytosolic proteins were analyzed by Western blotting with anti-active-β-catenin antibody. In (**B**) and (**C**), β-actin was used as a loading control, and β-catenin levels were normalized to those of β-actin. The bar graph indicates the average volume density corrected for the loading control, and results are expressed as the mean ± SD of three independent experiments. *p < 0.05 and **p < 0.01, comparison between the BIO-treated control and kinetin riboside-treated groups.

GSK-3β-mediated β-catenin phosphorylation at residues Ser33/37 is a prerequisite for proteasome-dependent β-catenin degradation via the Wnt/β-catenin signaling pathway. Therefore, we used 6-bromoindirubin-3’-oxime (BIO), an inhibitor of GSK-3β [21], to determine whether GSK-3β activity is required for the inhibitory effect of kinetin riboside on the Wnt/β-catenin pathway. When HEK293-FL reporter cells are treated with BIO, CRT becomes activated (Fig. 3A). Interestingly, kinetin riboside treatment abrogates BIO-mediated CRT activation (Fig. 3A). In addition, Western blot analysis consistently shows that the BIO-induced accumulation of intracellular β-catenin protein is attenuated after treating HEK293-FL reporter cells with kinetin riboside (Fig. 3B). These results indicate that kinetin riboside induces β-catenin degradation via a mechanism that is independent of GSK-3β.

### Kinetin Riboside Promotes β-Catenin Down-Regulation and Suppresses Target Gene Expression in Colorectal Cancer Cells

**Fig. 4:**
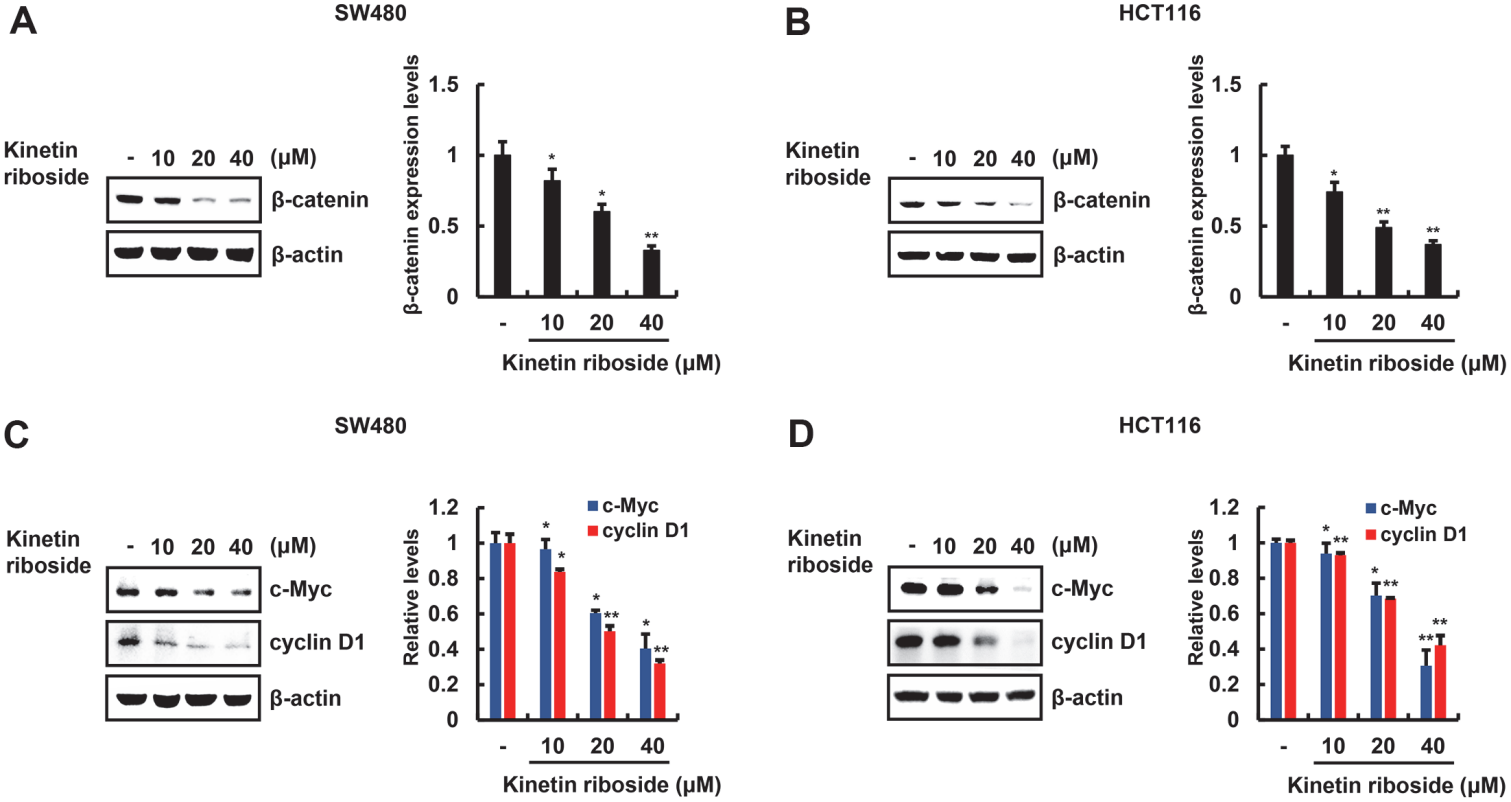
Kinetin riboside decreases the levels of β-catenin and its target genes in colorectal cancer cells. (**A, B**) SW480 and HCT116 cells were incubated with vehicle (DMSO) or kinetin riboside (10, 20, or 40 μM) for 15 h, and cytosolic proteins were analyzed by Western blotting with anti-β-catenin antibody. (**C, D**) Whole-cell extracts were prepared from SW480 and HCT116 cells incubated for 48 h, and proteins were analyzed by Western blotting with anti-cyclin D1 and anti-c- Myc antibodies. In (**A**) to (**D**), β-actin was used as a loading control, and β-catenin levels were normalized to those of β-actin. The bar graph indicates the average volume density corrected for the loading control, and results are expressed as the mean ± SD of three independent experiments. *p < 0.05 and **p < 0.01, comparison between the DMSO control and kinetin ribosidetreated groups.

We evaluated the effect of kinetin riboside on the levels of intracellular β-catenin protein in SW480 and HCT116 colon cancer cells by Western blot analysis. Upregulation of intracellular β-catenin in SW480 and HCT116 colon cancer cells occurs due to an inactivation mutation in the *APC* gene and a Ser45 (CK1 phosphorylation site) deletion mutation in β-catenin, respectively [22]. As depicted in Figs. 4A and 4B, kinetin riboside decreases intracellular β-catenin levels in a concentration-dependent manner in these cells. We next examined the effect of kinetin riboside on the expression of β-catenin-dependent genes in SW480 and HCT116 colon cancer cells. We found that the levels of c-Myc and cyclin D1 proteins, which play essential roles in cell proliferation, decrease in a concentration-dependent manner. These results indicate that kinetin riboside increases oncogenic β-catenin protein turnover in colon cancer cells.

### Kinetin Riboside Inhibits Colorectal Cell Proliferation

**Fig. 5:**
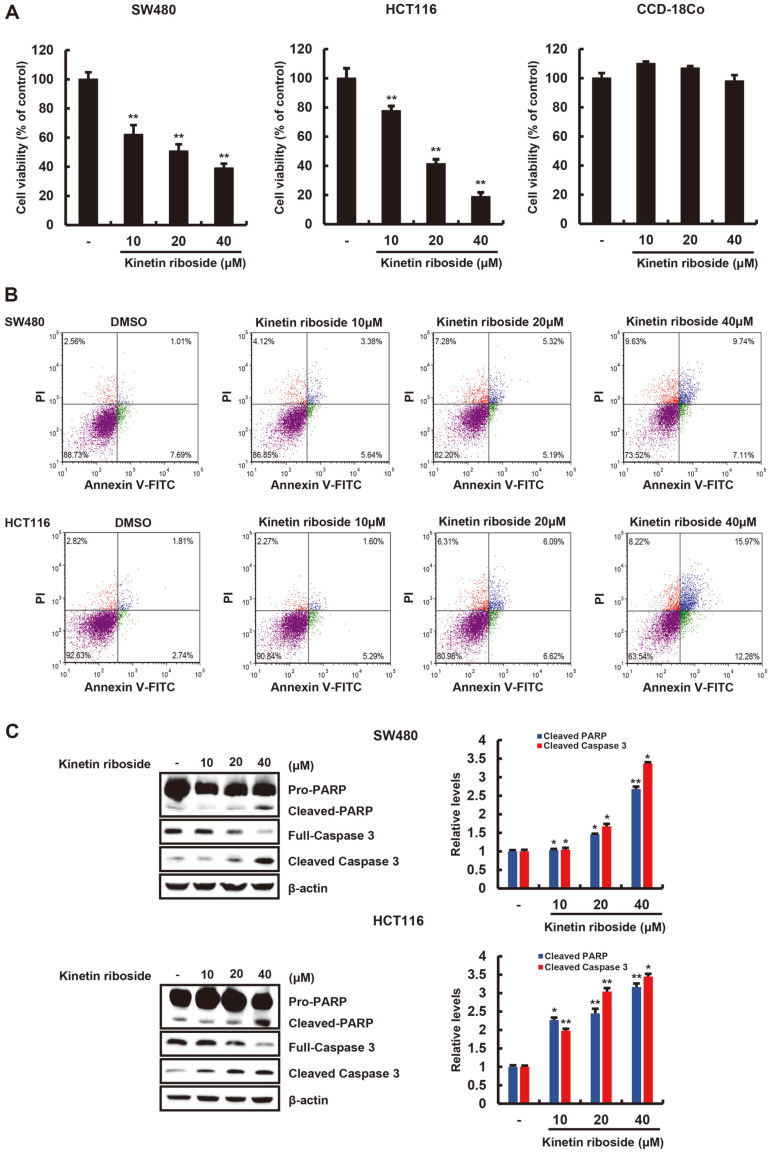
The effect of kinetin riboside on colorectal cancer cell growth. (**A**) SW480 and HCT116 cells were treated with kinetin riboside (10, 20, or 40 μM) for 48 h, and cell viability was measured using a CellTiter-Glo assay. (**B**) SW480 and HCT116 cells were incubated with vehicle (DMSO) or kinetin riboside (10, 20, or 40 μM) for 48 h and then stained with Annexin V-FITC and propidium iodide (PI). The x- and y-axes indicate annexin V-FITC intensity and PI fluorescence, respectively. (**C**) SW480 and HCT116 cells were incubated with vehicle (DMSO) or kinetin riboside (10, 20, or 40 μM) for 48 h, and whole-cell extracts were analyzed by Western blotting with anti-caspase-3, anti-cleaved caspase-3, and anti-poly (ADPriboside) polymerase (PARP).

Several studies have reported that a specific reduction of β-catenin suppresses the growth of colon cancer cells [23, 24]. Because kinetin riboside accelerates oncogenic β-catenin degradation, we investigated the effect of kinetin riboside on the growth of SW480 and HCT116 cells. As expected, kinetin riboside decreases the proliferation of both cell lines in a concentration-dependent manner (Fig. 5A). On the other hand, CCD-18Co, a normal cell of the colon, treated by kinetin riboside did not exhibit significant changes in the levels of cell viability. We performed apoptosis analysis in SW480 and HCT116 to evaluate whether kinetin riboside exerts any antiproliferative effects. When SW480 and HCT116 cells are treated with kinetin riboside, the number of apoptotic annexin V/PI double-positive cells increases from 8.70% to 16.85% in SW480 cells and from 4.55% to 28.25% in HCT116 cells (Fig. 5B). Moreover, Western blot analysis showed that kinetin riboside forms active caspase-3 (cleaved form) and induced proteolytic cleavage of poly (ADP-riboside) polymerase (PARP), biochemical markers of apoptosis (Fig. 5C). In addition, treatment of SW480 and HCT116 cell with kinetin riboside increased the levels of the proapoptotic protein Bax, a core regulator of the intrinsic pathway of apoptosis (Fig. S2). These results suggest that kinetin riboside suppresses colon cancer cell growth by inducing the intrinsic pathway of apoptosis.

## Discussion

Kinetin riboside displays antiproliferative and apoptogenic activities in various human cancer cell lines. It has recently been shown to suppress tumor growth in murine xenograft models of human leukemia and melanoma [25, 26]. However, the mechanisms by which kinetin riboside induces growth inhibition of colon cancer cells have not been elucidated. In this study, we demonstrated that kinetin riboside down-regulates β-catenin through a GSK-3β-independent proteasomal degradation pathway.

The stability of intracellular β-catenin protein is typically controlled by a destruction complex (APC/Axin/CK1/GSK-3β)-dependent pathway. N-terminal phosphorylation of β-catenin within a destruction complex is a prerequisite for the degradation of β-catenin through a proteasome-dependent mechanism [5, 6]. However, several lines of evidence from the present study suggest that kinetin riboside promotes β-catenin degradation through a mechanism that does not involve a destruction complex. First, kinetin riboside induces degradation of β-catenin in HCT116 colon cancer cells, which express β-catenin proteins mutated at the CK1 phosphorylation site (Ser45), suggesting that CK1 is not involved in kinetin riboside-mediated β-catenin degradation. Second, in the presence of BIO, a pharmacological inhibitor of GSK-3β, intracellular β-catenin was still degraded by kinetin riboside treatment, indicating that GSK-3β activity is not required for β-catenin down-regulation by kinetin riboside. Finally, kinetin riboside induces β-catenin degradation in SW480 cells, which harbor a mutation in APC, suggesting that β-catenin degradation is independent of APC. Similarly, activation of protein kinase Cα with CGK062 has been reported to increase β-catenin turnover by a destruction complex-independent mechanism [27].

Natural compounds that suppress the proliferation of colon cancer cells through attenuation of the Wnt/β-catenin pathway have been identified. Genestein, a leading representative of the isoflavone family, inhibits the Wnt/β-catenin pathway by increasing a Wnt antagonist sFRP2 gene expression in DLD-1 colon cancer cells [28]. Resveratrol disrupts β-catenin/TCF interaction, thereby inhibiting the Wnt/β-catenin pathway, in colon cancer cells [29]. From our previous study, curcumin, a component of turmeric, inhibits the growth of colon cancer cells by suppressing Wnt/β-catenin signaling through down-regulation of the p300 coactivator [30]. Quercetin, which belongs to the flavonol family, attenuate Wnt/β-catenin signaling by decreasing nuclear β-catenin and Tcf-4 expression in SW480 colon cancer cells [31]. In the present study, we observed that kinetin riboside-mediated β-catenin degradation was nullified by treatment of MG-132, a proteasome inhibitor, indicating that kinetin riboside suppresses Wnt/β-catenin signaling via proteasomal degradation of β-catenin.

In conclusion, we showed that kinetin riboside is a Wnt/β-catenin pathway antagonist and demonstrated its antiproliferative activity against colorectal cancer cells. Kinetin riboside promotes intracellular β-catenin degradation via a mechanism that does not involve the β-catenin destruction complex. It also inhibits the proliferation of colorectal cancer cells by inducing apoptosis. These findings suggest that kinetin riboside can potentially serve as a preventive or therapeutic agent against cancers involving abnormal β-catenin accumulation.

## Supplemental Materials

Supplementary data for this paper are available on-line only at http://jmb.or.kr.
