# Supplementary material for: Apoptosis of Kinetin Riboside in Colorectal Cancer Cells Occurs by Promoting β-Catenin Degradation
Source: J Microbiol Biotechnol. 2023 Jun 23;33(9):1206–12. doi: 10.4014/jmb.2301.01035 (PMC10580898; doi:10.4014/jmb.2301.01035)
Supplement: Supplementary file 1 [file jmb-33-9-1206-supple.pdf]

J. Microbiol. Biotechnol.
https://doi.org/10.4014/jmb.2301.01035

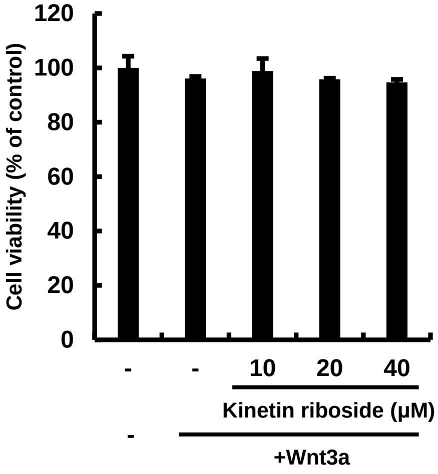

Supplementary Fig. S1. HEK293-FL cells were incubated for 15 h with vehicle (DMSO) or the indicated concentrations of kinetin riboside (10, 20, or 40 μM) in the presence of Wnt3a-CM and cell viability was measured using a CellTiter-Glo assay (Promega). Results are expressed as the mean ± SD of three independent experiments.

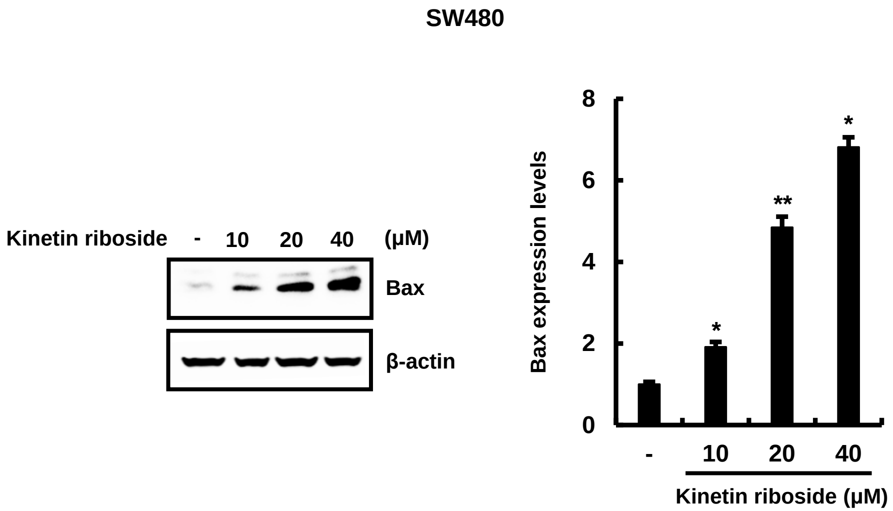

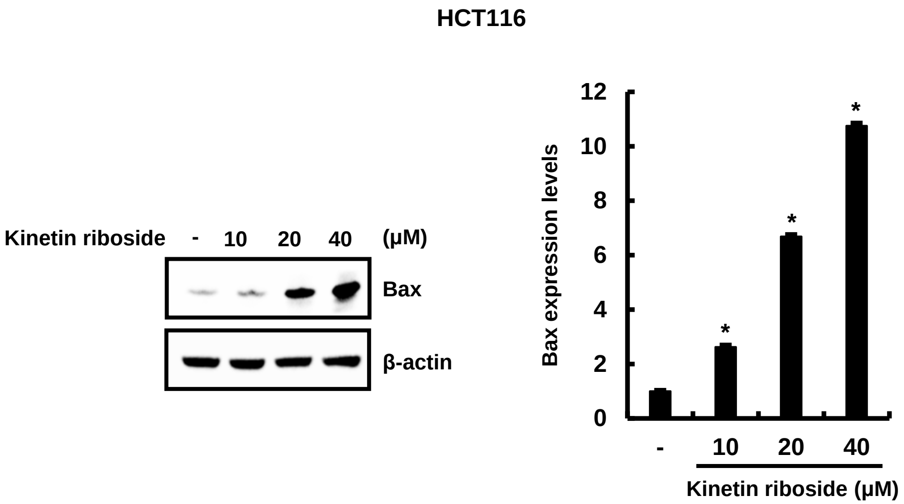

Supplementary Fig. S2. SW480 and HCT116 cells were treate with vehicle (DMSO) or kinetin riboside (10, 20, or 40 μM) for 48 h and then subject to western blot analysis using an anti-Bax antibody. The bar graph indicates the average volume density corrected for the loading control, and results are expressed a s the mean ± SD of three independent experiments. *P < 0.05 and **P < 0.01, comparison between the DMSO control and kinetin riboside-treated groups.
